# Differences in Diffusion Tensor Imaging White Matter Integrity Related to Verbal Fluency Between Young and Old Adults

**DOI:** 10.3389/fnagi.2021.750621

**Published:** 2021-11-22

**Authors:** Benjamin Yeske, Jiancheng Hou, Nagesh Adluru, Veena A. Nair, Vivek Prabhakaran

**Affiliations:** ^1^Department of Radiology, School of Medicine and Public Health, University of Wisconsin–Madison, Madison, WI, United States; ^2^Center for Cross-Strait Cultural Development, Fujian Normal University, Fuzhou, China; ^3^Waisman Center, University of Wisconsin–Madison, Madison, WI, United States; ^4^Department of Psychology, Department of Psychiatry, University of Wisconsin–Madison, Madison, WI, United States; ^5^Neuroscience Training Program, University of Wisconsin–Madison, Madison, WI, United States

**Keywords:** diffusion tensor imaging (DTI), aging, tract-based spatial statistics (TBSS), white matter integrity, verbal fluency

## Abstract

Throughout adulthood, the brain undergoes an array of structural and functional changes during the typical aging process. These changes involve decreased brain volume, reduced synaptic density, and alterations in white matter (WM). Although there have been some previous neuroimaging studies that have measured the ability of adult language production and its correlations to brain function, structural gray matter volume, and functional differences between young and old adults, the structural role of WM in adult language production in individuals across the life span remains to be thoroughly elucidated. This study selected 38 young adults and 35 old adults for diffusion tensor imaging (DTI) and performed the Controlled Oral Word Association Test to assess verbal fluency (VF). Tract-Based Spatial Statistics were employed to evaluate the voxel-based group differences of diffusion metrics for the values of fractional anisotropy (FA), mean diffusivity (MD), axial diffusivity (AD), radial diffusivity (RD), and local diffusion homogeneity (LDH) in 12 WM regions of interest associated with language production. To investigate group differences on each DTI metric, an analysis of covariance (ANCOVA) controlling for sex and education level was performed, and the statistical threshold was considered at *p* < 0.00083 (0.05/60 labels) after Bonferroni correction for multiple comparisons. Significant differences in DTI metrics identified in the ANCOVA were used to perform correlation analyses with VF scores. Compared to the old adults, the young adults had significantly (1) increased FA values on the bilateral anterior corona radiata (ACR); (2) decreased MD values on the right ACR, but increased MD on the left uncinate fasciculus (UF); and (3) decreased RD on the bilateral ACR. There were no significant differences between the groups for AD or LDH. Moreover, the old adults had only a significant correlation between the VF score and the MD on the left UF. There were no significant correlations between VF score and DTI metrics in the young adults. This study adds to the growing body of research that WM areas involved in language production are sensitive to aging.

## Introduction

Throughout adulthood, the brain undergoes an array of structural and functional changes during the typical aging process (Caserta et al., 2009). These changes involve decreased brain volume, reduced synaptic density, and alterations in white matter (WM; Masliah et al., 1993; Jernigan et al., 2001; Resnick et al., 2003). The structural deterioration of the brain is thought to be the reason for cognitive decline seen in the aging process; therefore, correlational studies comparing changes to brain structure and function are increasingly common. These neuroimaging studies have repeatedly shown age-related cortical network re-organization, specifically a reduction of hemispheric specialization toward more bilateral activation. This reduction of hemispheric specialization, known as the hemispheric asymmetry reduction in older adults (HAROLD) model (Cabeza, 2002), is well documented in studies using various imaging modalities, namely, electro-encephalography (Bellis et al., 2000), near-infrared spectroscopy (Herrmann et al., 2006), functional magnetic resonance imaging (fMRI; Cabeza, 2002; La et al., 2016), and diffusion tensor imaging (DTI; Ardekani et al., 2007). In addition to the reduction of hemispheric specialization, an anteroposterior gradient of the loss of WM integrity has also been observed, with the anterior regions of the brain being disproportionately affected in the aging process compared to the posterior regions (Pfefferbaum et al., 2005; Ardekani et al., 2007; Madden et al., 2009; Bennett et al., 2010; Sullivan et al., 2010).

Extensive research has been conducted to explore the aging declines seen in cognitive abilities, namely, working memory (Nyberg et al., 2012; Vaqué-Alcázar et al., 2020), executive function (Fjell et al., 2017; Webb et al., 2020), and language function (Wingfield and Grossman, 2006; Kantarci et al., 2011; Kemmotsu et al., 2012; Baciu et al., 2016). Previous neuroimaging studies concerning language function have measured the ability of adult language production and its correlations to brain function (Pihlajamäki et al., 2000), used fMRI to study language function (Wingfield and Grossman, 2006; Meinzer et al., 2009; Baciu et al., 2016), and studied structural gray matter volume involved in language function (Zhang et al., 2013); however, the structural role of WM in adult language production in individuals across the life span remains to be thoroughly elucidated.

One non-invasive MRI technique for *in vivo* mapping of the structures of WM is DTI, which provides detailed information on the underlying fiber tract architecture as reflected by diffusion patterns of water molecules (Sundaram et al., 2008; Pugliese et al., 2009). Fractional anisotropy (FA, a scalar measure of the directional constraint of water diffusion) and mean diffusivity (MD, the mean of three eigenvectors that each reflects separate directions of minimal and maximal diffusion) are the most frequently used metrics to investigate WM fiber tract integrity. More recently, studies on aging have included axial diffusivity (AD, a scalar measure of diffusivity along the length of an axon; see Thomason and Thompson, 2011) and radial diffusivity (RD, measure of water diffusion perpendicular to the axons and is associated with demyelination and neuro-inflammation with edema and macrophage infiltration; see Budde et al., 2011; Rayhan et al., 2013) in their analyses, as they are more specific to neural changes commonly involved in aging, namely, axonal damage or loss (AD; Song et al., 2003; Budde et al., 2007) and the degree of myelination (RD) (Song et al., 2002, 2003, 2005; Nair et al., 2005; Budde et al., 2007). Using these two metrics, some patterns have been identified to describe the differential aging associations in WM fiber tracts. For example, in some WM tracts, age-related decreases in FA are associated with increases only in RD, but not in AD (Bhagat and Beaulieu, 2004; Davis et al., 2009; Madden et al., 2009; Zhang et al., 2010). Other patterns observed are that decreases in FA are associated with significant increases in both RD and AD (Sullivan and Pfefferbaum, 2006; Zahr et al., 2009; Sullivan et al., 2010) and decreases in FA are associated with increases in RD and decreases in AD (Bennett et al., 2010). These patterns suggest that there may be differential aging processes occurring in different brain regions. Currently, these patterns remain unstudied in their relationship to age-related language production. Additionally, a novel inter-voxel metric called local diffusion homogeneity (LDH), which quantifies the local coherence of water molecule diffusion in a model-free manner, was also examined in our analyses (Gong, 2013). Using the LDH metric to describe the WM fiber tracts is still in its infancy and several studies have reported it as being complementary to FA and MD in detecting changes in WM (Gong, 2013; Liu et al., 2017; Liang et al., 2019).

Currently, there are no studies using LDH to assess language production or brain aging more broadly; however, there are a handful of studies that have taken advantage of other DTI metrics to assess the structural role of WM in typically aging adults and language production. Stamatakis et al. (2011) identified that FA of the superior longitudinal fasciculus (SLF) and inferior longitudinal fasciculus (ILF) was positively correlated with accuracy in naming famous individuals. Madhavan et al. (2014) observed increased FA values on the SLF and increased age were positively associated with the performance in verbal fluency (VF) and word retrieval, respectively. They also identified a relationship between gender and FA values on the SLF tract and reported a linear decrease in FA in males and increase in FA in females until age 40, followed by a gradual decline. Houston et al. (2019) reported the performance on a word-retrieval task was associated with increased FA within the inferior fronto-occipital fasciculus (IFOF), in addition to the SLF, and observed increased FA within the corpus callosum that was associated with lower VF scores. Troutman and Diaz (2020) observed among all adult age groups that better performance on a naming with distractors task was associated with lower RD across dorsal, ventral, and fronto-striatal tracts as well as higher FA along dorsal tracts but was unable to find an association when covarying for age groups. Teubner-Rhodes et al. (2016) looked at age-related performance in acquisition and retrieval of lexical and semantic information and found age-related declines in arcuate fasciculus (AF) microstructure were related to cognitive processing speed, but not to vocabulary retrieval. Interestingly, FA on the left AF was significantly related to individual variability in vocabulary independent of age, suggesting that the orientation and organization of the AF tract are stable with aging (Teubner-Rhodes et al., 2016). Taken together, these studies suggest that the dorsal stream pathway, specifically the SLF, may have a significant contribution to age-related differences in language production, but further research is needed.

Other tracts have also been identified to be key language comprehension and production pathways by studying patients suffering from diseases that afflict language function, namely, aphasia and strokes. In these patient populations, several WM tracts have been identified for being involved in language comprehension, namely, the IFOF (Ivanova et al., 2016; Hula et al., 2020), uncinate fasciculi (UF; Hula et al., 2020), middle longitudinal fasciculi (Hula et al., 2020), corona radiata (Grönholm et al., 2016; Sul et al., 2019), and external capsule (EC; Chen et al., 2015), and in language production, namely, the AF (Ivanova et al., 2016), middle longitudinal fasciculi (Hula et al., 2020), corona radiata (Grönholm et al., 2016; Sul et al., 2019), and EC (Chen et al., 2015). It is evident that injury to the aforementioned areas is known to affect language function; however, it is less understood how these regions are affected by the typical aging process and what effect this process has on language function.

This study employs Tract-Based Spatial Statistics (TBSS) to examine the regional brain differences related to language function (see the “Region of Interest Selection” in the “Materials and Methods” section for detailed regions) between young and old adults. TBSS is applied to perform automated analysis of WM integrity. TBSS uses a fine-tuned non-linear registration method followed by a projection onto a mean FA skeleton. This skeleton represents the centers of all tracts common to the group and the resulting data fed into voxel-wise cross-subject statistics. Thus, TBSS combines the strength of both voxel-based and tractographic analyses to overcome the limitations of conventional methods, namely, standard registration algorithms and spatial smoothing (Dunst et al., 2014). Moreover, TBSS is assumed to improve the sensitivity, objectivity, and interpretability of multi-subject diffusion imaging studies (Smith et al., 2006; Dunst et al., 2014).

The VF task, which has traditionally been administered as a clinical neuropsychological paradigm to assess linguistic and executive function abilities, was used as behavioral testing in this study; it is one of the most widely used paradigms because of its simplicity and ease of administration. The cognitive components assessed by the VF task include executive functions, namely, initiation, inhibition, planning, updating, and shifting as well as verbal long-term memory (word knowledge) and lexical–semantic linguistic processes (Shao et al., 2014).

Using group differences seen on WM metrics (i.e., FA, MD, AD, RD, and LDH) in old vs. young adults, as well as group performance on the VF task, we hope to elucidate the impact aging has on the WM integrity and its relationship to language function. Portions of this manuscript have been previously presented (Hou et al., 2018).

## Materials and Methods

### Participants

Notably, 38 young adults (21 men and 17 women, mean age = 23.58 ± 3.35 years) and 35 old adults (19 men and 16 women, mean age = 60.91 ± 5.25 years) were recruited from the Madison, Wisconsin, campus community. They were free of any medical, neurological, or psychiatric disorders and had at least 14 years of education. A subset of the participants (*n* = 44) received the Mini Mental State Examination (MMSE; Folstein et al., 1975) and had scores ≥29. For participants with educational level of high school graduate, a score on the MMSE of ≤25 was considered cognitively impaired. The Edinburgh Handedness Inventory (Oldfield, 1971) was administered to all participants. A score greater than +40 was considered right-handed, between −40 and +40 was considered ambidextrous, and less than −40 was considered left-handed. Based on these criteria, there were 64 right, 7 left, and 2 ambidextrous in the study sample. Table 1 provides the basic demographic information of participants. All participants provided written informed consent. The experimental protocols were approved by the Institutional Review Board (IRB) of the School of Medicine and Public Health, University of Wisconsin–Madison.

**TABLE 1:** Demographic data and differences of VF testing.

Characteristics	Young	Old	*t*	*p*
Number	38	35		
Age (years) [range]	23.58 (3.35) [18–32]	60.91 (5.25) [55–77]	28.64	0.000
Education (years) [range]	16.79 (2.22) [12–21]	17.43 (2.87) [12–22]	1.068	0.289
Sex (male/female)	21/17	19/16	0.678	0.878
VF z-score	−0.28 (1.03)	0.35 (1.23)	2.355	0.021
Handedness (right/left/amb)	33/3/2	31/4/0	8.574	0.127

*Standard deviations are shown in parentheses. VF, verbal fluency; amb, ambidextrous.*

### Behavioral Testing

We administered the phonemic VF task [the Controlled Oral Word Association Test (COWAT); Benton and Hamsher, 1976] to test cognitive function. The COWAT has been extensively used in both clinical and non-clinical populations because of its face validity (Sauzéon et al., 2011), assessment of both verbal cognitive ability and executive control (Fisk and Sharp, 2004), and high correlation with measures of attention, verbal memory, and word knowledge (Ruff et al., 1997). Participants were required to produce words beginning with the letters “F,” “A,” and “S” in three 1-min trials, respectively. Raw VF scores were based on the total correct responses over the three trials, which were then used to compute age and education corrected VF z-scores based on a normative database (Tombaugh et al., 1999). This corrected VF z-score was used to quantify performance of VF for each participant.

### Magnetic Resonance Imaging Data Acquisition

Diffusion-weighted images were acquired using a spin-echo based, single-shot, echo-planar diffusion sequence lasting 10 min on a GE750 3 T MRI scanner. The specific parameters of MRI were as follows: repetition time (TR) = 9,000 ms; echo time (TE) = 76.6 ms; single average (NEX = 1); field of view = 100 mm × 100 mm; matrix size = 256 × 256; in-plane resolution = 1 mm × 1 mm; 75 axial slices with no gap between slices and slice thickness = 2 mm; excitation flip angle α = 90°; 56 gradient encoded directions *b*-value = 1,000 s/mm^2^, 10 volumes with *b*-value = 0 s/mm^2^. A high-resolution three-dimensional T1-weighted BRAVO, IR-prepared Fast Spoiled Gradient Echo (FSPGR), MRI sequence with 156 axial slices was performed for each participant using the following parameters: TR = 8.132 ms; TE = 3.18 ms, inversion time (TI) = 450 ms; field of view = 256 mm × 256 mm; matrix size = 256 × 256; in-plane resolution = 1 mm × 1 mm; slice thickness = 1.0 mm; excitation flip angle α = 12°.

### Data Preprocessing

All diffusion data were processed using the “Pipeline for Analyzing braiN Diffusion images” (PANDA): a toolbox implemented in MATLAB^1^ (Cui et al., 2013). This software employs several neuroimaging processing modules, namely, the FMRIB Software Library (FSL), the Pipeline System for Octave and Matlab (PSOM), the Diffusion Toolkit, and the MRIcron to automatically perform a series of steps (i.e., skull removal, correction of eddy current distortion, build diffusion tensor models) (Cui et al., 2013; Kashfi et al., 2017; Hou et al., 2020).

**FIGURE 1:**
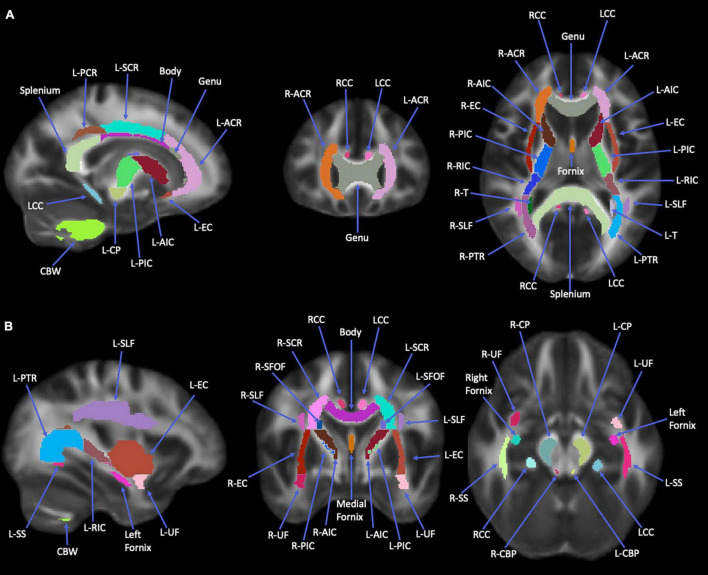
Images of Johns Hopkins University (JHU)-ICBM-DTI-81 white matter (WM) atlas. This atlas was used to obtain the 12 regions of interest used in study analyses. Images are all WM tracts available in this atlas. **(A)** Orientations from left to right: sagittal, coronal, axial. **(B)** Orientations from left to right: sagittal, coronal, axial. Splenium, splenium of corpus callosum; Body, body of corpus callosum; Genu, genu of corpus callosum; L-ACR, left anterior corona radiata; R-ACR, right anterior corona radiata; L-SCR, left superior corona radiata; L-PCR, left posterior corona radiata; RCC, right cingulum cortex; LCC, left cingulum cortex; L-EC, left external capsule; L-AIC, left anterior limb of internal capsule; R-AIC, right anterior limb of internal capsule; L-PIC, left posterior limb of internal capsule; R-PIC, right posterior limb of internal capsule; L-RIC, left retrolenticular part of internal capsule; R-RIC, right retrolenticular part of internal capsule; L-CP, left cerebral peduncle; L-PTR, left posterior thalamic radiation; R-PTR, right posterior thalamic radiation; L-T, left tapetum; R-T, right tapetum; CBW, cerebellar white matter; L-UF, left uncinate fasciculus; R-UF, right uncinate fasciculus; R-EC, right external capsule; L-SLF, left superior longitudinal fasciculus; L-SS, left sagittal stratum; R-SS, right sagittal stratum; R-SCR, right superior corona radiata; L-SFOF, left superior fronto-occipital fasciculus; R-SFOF, right superior fronto-occipital fasciculus; R-CP, right cerebral peduncle; L-CBP, left cerebellar peduncle; R-CBP, right cerebellar peduncle. The regional masks were filtered with the white matter skeleton mask from TBSS (see Figure 2).

**FIGURE 2:**
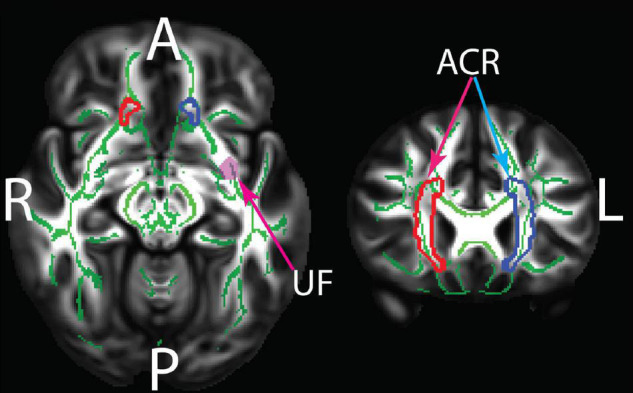
The JHU white matter regions showing statistically significant group differences between old and young adults. ACR, anterior corona radiata; UF, uncinate fasciculus. The underlay shows the TBSS white matter skeleton which was masked by the JHU regions in the statistical analyses. The TBSS skeleton and the JHU regions are overlaid on the HCP 1065 FA template. Green: White matter skeleton; Red: right ACR; Blue: left ACR; pink: left UF.

Diffusion metrics such as FA, MD, AD, RD (Smith et al., 2006; Smith and Nichols, 2009; Cui et al., 2013), and LDH (Gong, 2013) for each participant were extracted for 50 tracts identified from the Johns Hopkins University (JHU)-ICBM-DTI-81 WM atlas (Mori et al., 2008; Cui et al., 2013). Figures 1, 2 illustrate the representative images of WM tracts for this atlas. These region masks were filtered by applying the TBSS WM skeleton. Each global mean metric (i.e., FA, MD, AD, RD, and LDH) for each participant was obtained by averaging across the 50 labels, with the diffusion metric for each label being divided by this global mean to account for any variability between participants. This standardized metric was used in our statistical analysis.

### Region of Interest Selection

Based on prior studies on WM organization of the brain, six WM labels in each hemisphere implicated in language function (Smits et al., 2014; Friederici, 2015) were selected as regions of interests (ROIs), namely, ACR, superior corona radiata (SCR), posterior corona radiata (PCR), EC, UF, and SLF.

### Statistical Analysis

A chi-square test was performed for handedness group differences, which did not identify a statistically significant difference between the two groups (see Table 1 for details). As a result, all handedness variations (right, left, and ambidextrous) were kept in the participant sample.

To investigate group differences on each DTI metric, an analysis of covariance (ANCOVA) controlling for sex and educational years was performed, and the statistical threshold for significance was considered at *p* < 0.00083 (0.05/60 labels) after Bonferroni correction for multiple comparison. This analysis identified tracts in which the diffusivity metrics were significantly different between the groups. Pearson’s correlation analysis, with sex as a covariate, was performed between the diffusivity metrics in the significant tracts and VF scores using IBM SPSS version 27 and considered significant at uncorrected *p* < 0.05.

## Results

**FIGURE 3:**
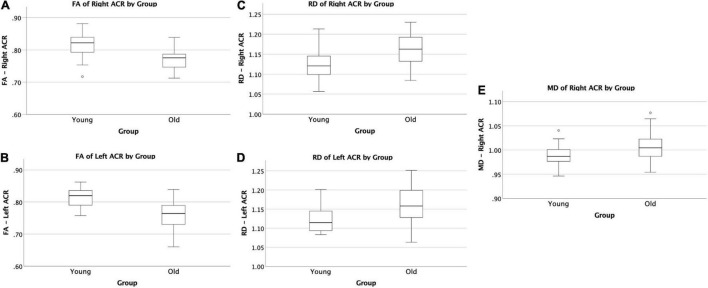
Significant difference in ACR by group comparisons. **(A)** Boxplot showing significant reduction in FA in old adults compared to young adults on the right ACR. **(B)** Boxplot showing significant reduction in FA in old adults compared to young adults on the left ACR. **(C)** Boxplot showing significant increase in RD in old adults compared to young adults on the right ACR. **(D)** Boxplot showing significant increase in RD in old adults compared to young adults on the left ACR. **(E)** Boxplot showing significant increase in MD in old adults compared to young adults on the right ACR. All relationships are considered at *p* < 0.00083 (Bonferroni multiple comparison 0.05/60 labels) after controlling for sex and education. FA, fractional anisotropy; RD, radial diffusivity; MD, mean diffusivity.

**FIGURE 4:**
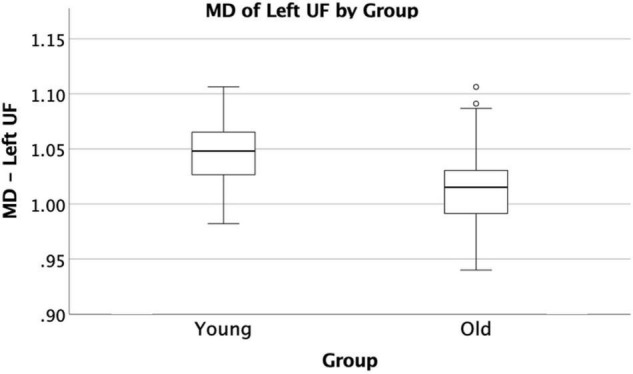
Significant difference in UF by group comparison. Boxplot showing significant decrease in MD in old adults compared to young adults on the left UF. All relationships are considered at *p* < 0.00083 (Bonferroni multiple comparison 0.05/60 labels) after controlling for sex and education.

**FIGURE 5:**
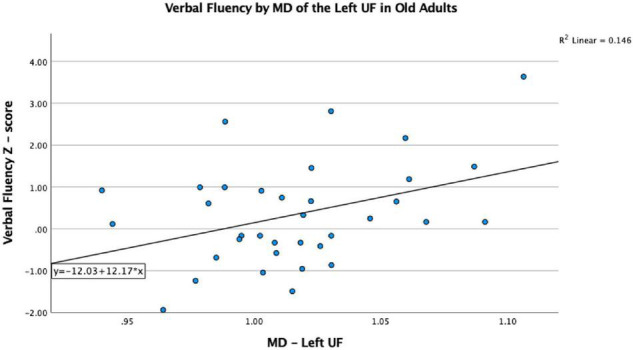
Significant correlation between VF and MD on the left UF in older adults, after controlling for sex, *p* < 0.05.

The VF testing demonstrated old adults have significantly higher VF z-scores than young adults (see Table 1). Compared to the old adults, the young adults had significantly (1) increased FA values on the bilateral ACR; (2) increased MD value on the left UF; and (3) decreased RD on the bilateral ACR. There were no significant differences between the groups for AD or LDH (Table 2 and Figures 3, 4). Moreover, the old adults had only a significant positive correlation between the VF z-score and the MD on the left UF [*r*_(35)_ = 0.383, *p* = 0.025], with sex as a covariate (see Figure 5). There were no significant correlations between VF score and DTI metrics in young adults.

**TABLE 2:** Significant difference between DTI metrics and tract by group comparisons.

	Tracts	Mean difference (young–old)	Standard error	*p*	95% confidence interval for difference	
					Lower bound	Upper bound
FA	Right ACR	0.0454	0.0080	0.0000	0.0175	0.0732
	Left ACR	0.0511	0.0083	0.0000	0.0221	0.0802
MD	Right ACR	–0.195	0.0053	0.0005	–0.0380	–0.0009
	Left UF	0.0291	0.0078	0.0004	0.0018	0.0564
RD	Right ACR	–0.0415	0.0084	0.0000	–0.0709	–0.0121
	Left ACR	–0.0424	0.0094	0.0000	–0.0752	–0.0097

*All DTI metric values are *p* < 0.00083 (Bonferroni multiple comparison 0.05/60 labels) after controlling for sex and education. FA, fractional anisotropy; MD, mean diffusivity (unit: μm^2^/ms); RD, radial diffusivity (unit: μm^2^/ms); ACR, anterior corona radiata; UF, uncinate fasciculus.*

## Discussion

As reported previously in the literature, our study identified age-related reductions in FA and increases in RD on the bilateral ACR as well as an increase in MD on the right ACR (Barrick et al., 2010; Bennett et al., 2010; Ly et al., 2014). Additionally, MD on the left UF showed significant group differences adding to the growing body of research that WM areas indicated in language function are sensitive to aging.

Hemispheric asymmetry with aging is well known (Cabeza, 2002). Specifically, the left lateralized language pattern seen in young adults changes with age to a more bi-hemispheric pattern in older adults, which could be a compensatory mechanism to maintain behavioral performance. This study observed decreased MD on the left UF and increased MD on the right ACR in the old adults group, suggesting an improvement of the UF integrity and a decrease in the ACR. However, a decrease in left UF integrity would be predicted by the HAROLD model if the left hemispheric predominance of language function were to be reduced toward more bilateral activation (Cabeza, 2002). While this finding may seem to contradict the HAROLD model, it is difficult to assess the reduction of hemispheric specialization with only two non-bilateral group differences. Additionally, in this study, we only included one test of language/executive function. The asymmetry pattern is best investigated using a specific battery of language tests and will be explored in a future study.

This study did find evidence for another model of the aging brain, the anteroposterior model. Since the ACR resides more anteriorly than the other brain regions we studied, our findings for the ACR agree with prior evidence for the existence of an anteroposterior model for loss of WM integrity in the aging brain (Pfefferbaum et al., 2005; Ardekani et al., 2007; Madden et al., 2009; Bennett et al., 2010). Since the UF is located inferiorly to the ACR, our findings of increased MD on the UF in old adults compared to young adults seem to contradict this assertion of anteroposterior aging. However, there is evidence to suggest that superiorly located fiber systems demonstrate age effects earlier than inferior systems (Sullivan et al., 2010) and this finding is additional evidence that there are brain regions with differential aging processes in aging adults. In addition, the significant increase in RD on the ACR signifies an age-related demyelination effect of that tract, based on previous animal and human studies investigating RD and its relationship to neural networks (Song et al., 2002, 2003, 2005; Nair et al., 2005; Budde et al., 2007), as well as evidence supporting typical aging is accompanied by myelin damage and loss (Peters, 2002). Our study did not find any association between ACR and VF, but Troutman and Diaz (2020) observed that higher RD across dorsal, ventral, and fronto-striatal tracts was associated with poorer performance on a naming with distractors task, suggesting that demyelination can result in poorer language performance.

Compared to young adults, the significantly higher VF score we observed in the old adults group, in conjunction with the age-related demyelination and loss of WM integrity, could be evidence for the use of compensatory strategies to maintain performance. For instance, the HAROLD model purports that certain cognitive functions, namely, language production (La et al., 2016; Kim et al., 2018), lose their hemispheric specialization as one ages to counteract age-related neurocognitive deficits (Cabeza et al., 1997; Cabeza, 2002). Additionally, the compensation view of the HAROLD model has evidence to support recovery of language function after brain injuries (Cao et al., 1999) and resections (Jehna et al., 2017). Studies looking at temporal lobe resections in patients with epilepsy observed post-operative increases in FA in ipsilateral WM regions, such as the corona radiata and external and internal capsule, that were associated with a smaller fall in language proficiency after surgery (Yogarajah et al., 2010; Pustina et al., 2014). While age-related WM alterations may be different than the plastic changes occurring as a compensatory mechanism after regional brain insults, these studies still provide evidence that the brain has the capacity to rewire language pathways. Knowing this, perhaps age-related WM structural changes have a rewiring process to compensate for WM structures prone to degeneration allowing for preserved language function.

In our study, MD on the left UF of the old adult group was positively correlated with VF score, which is in contrast to what we would predict, since a higher MD typically indicates less WM integrity, and less WM integrity typically results in worse tract function. As a group, old adults have lower MD than young adults; however, within the old adult group, it is those with the higher MD values that score better on the VF task. Perhaps this indicates that the UF is becoming better organized to perform this task as we age, but only to a certain threshold, and perhaps the increased MD value in the older adults resulting in better performance is an indication of better compensation in those individuals by other WM tracts involved in VF performance. While we did not find other tract associations with the VF score that could attest to this postulation, Madhavan et al. (2014) did demonstrate a relationship with increased FA on the SLF and stronger language function in an aging population, suggesting some compensatory changes occurring in that tract. However, without other tract associations from this study, it is difficult to ascertain what the explanation for this may be, but future analyses involving a larger sample size may help elucidate this finding.

As previously mentioned, the demyelination and loss of WM integrity in the ACR, in addition to these UF findings, provides evidence that there may be differential aging processes in various WM tracts involved in language production that preserves this function with age. However, a few studies contradict this assertion by reporting losses of WM integrity in the UF with increasing age (Stamatakis et al., 2011; Kemmotsu et al., 2012; Gong, 2013). Taking this into account, it is difficult to interpret considering the sample sizes of the contradictory studies are all smaller than our current study. Further research will be needed to help resolve the discrepancy.

We did not identify any group differences for the AD and LDH DTI metrics, nor correlations of the VF score with the ACR, nor observe any significant group differences in the additional tracts we identified *a priori*. The lack of associations could have been a result of our small sample size and repeating this study with a larger population could elucidate more findings. While LDH is sensitive to diffusion properties among neighboring voxels and offers complementary information to FA, MD, and RD, for this study, it is possible that the metric is not as sensitive to the brain aging process (Sullivan et al., 2001; Pfefferbaum et al., 2005; Sullivan and Pfefferbaum, 2006; Westlye et al., 2010; Lamar et al., 2014; Madhavan et al., 2014; Houston et al., 2019; Troutman and Diaz, 2020).

## Data Availability Statement

The original contributions presented in the study are included in the article/supplementary material, further inquiries can be directed to the corresponding author/s.

## Ethics Statement

The studies involving human participants were reviewed and approved by the Institutional Review Board (IRB) of the School of Medicine and Public Health, University of Wisconsin–Madison. The patients/participants provided their written informed consent to participate in this study.

## Author Contributions

VP conceived and designed the experiments. VN helped with data acquisition. JH preprocessed the data and wrote the Materials and Methods section of the manuscript. BY and JH analyzed the data. BY wrote the Introduction, Results, and Discussion sections of the manuscript. VN, NA, and VP provided guidance for data analysis, and manuscript writing and editing. All authors contributed to the article and approved the submitted version.

## Conflict of Interest

The authors declare that the research was conducted in the absence of any commercial or financial relationships that could be construed as a potential conflict of interest.

## Publisher’s Note

All claims expressed in this article are solely those of the authors and do not necessarily represent those of their affiliated organizations, or those of the publisher, the editors and the reviewers. Any product that may be evaluated in this article, or claim that may be made by its manufacturer, is not guaranteed or endorsed by the publisher.

## Footnotes

1
http://www.nitrc.org/projects/panda/
